# Announcement of the Registered Report “Can a Variant of the Implicit Association Test Detect Nonsuicidal Self-Injury in a Clinical Population? A Registered Report”

**DOI:** 10.32872/cpe.11499

**Published:** 2023-03-31

**Authors:** Femke Cathelyn, Tilia Linthout, Pieter Van Dessel, Laurence Claes, Jan De Houwer

**Affiliations:** 1 Department of Experimental Clinical and Health Psychology, Ghent University, Ghent, Belgium; 2 Faculty of Psychology and Educational Sciences, University of Leuven, Leuven, Belgium; 3 Faculty of Medicine and Health Sciences, University of Antwerp, Antwerp, Belgium; Philipps-University of Marburg, Marburg, Germany

## Background

Nonsuicidal self-injury (NSSI) is a severe and prevalent mental health problem (Nock, 2010). Measures to detect which individuals are at risk for NSSI would be valuable for clinical practice. However, we still lack strong predictors of future NSSI behaviour, with the most notable exception being prior NSSI behaviour (Franklin et al., 2017; Griep & MacKinnon, 2022; Kiekens et al., 2018; Turner et al., 2013; Whitlock et al., 2013). Yet, the measurement of prior NSSI behaviour with self-report measures can be difficult because individuals may be motivated to conceal this harmful behaviour (Long, 2018; MacDonald et al., 2020; Simone & Hamza, 2020). To overcome this problem, an implicit measure has been developed that assesses automatic responding to statements about prior NSSI behaviour (i.e., the past nonsuicidal self-injury Implicit Association Test: P-NSSI-IAT; Cathelyn et al., 2021). Previous studies tested the predictive utility of this measure in online studies with samples of at risk participants and produced promising results (Franklin et al., 2017; Sohn et al., 2021).

## Aims

The main aim of this study is to validate the P-NSSI-IAT by assessing its ability to detect prior NSSI behaviour in a sample of clinical patients.

## Method

We will target patients who receive outpatient treatment for various conditions. Participants will first complete the P-NSSI-IAT. Next, they will be asked how many times they have intentionally cut or carved their skin without intending to kill themselves in the past twelve months and the past 30 days and how likely they would be to intentionally cut or carve their skin without intending to kill themselves in the future.

## Discussion

The registered study is the first to examine the clinical utility of a new implicit measure for prior NSSI behaviour (the P-NSSI-IAT). It will provide an answer to the question whether the P-NSSI-IAT allows detection of self-rated prior NSSI and future likelihood of NSSI in a sample of clinical patients.

## Supplementary Materials

The study protocol for this Registered Report is publicly accessible via PsychArchives.org (see Index of Supplementary Materials below).



CathelynF.
LinthoutT.
Van DesselP.
ClaesL.
De HouwerJ.
 (2023). Supplementary materials to "Announcement of the Registered Report “Can a variant of the Implicit Association Test detect nonsuicidal self-injury in a clinical population? A Registered Report”"
[Pre-registration protocol]. PsychOpen. 10.23668/psycharchives.12576


## Notes

**Editor's note:** This is an announcement of a Registered Report which received In-Principal-Acceptance (IPA) to be published in “Clinical Psychology in Europe”. The study protocol is publicly accessible at https://doi.org/10.23668/psycharchives.12576. In this announcement, a brief summary of the study protocol is presented.
